# The complete mitochondrial genomes of three *Ellobius* mole vole species (Rodentia: Arvicolinae)

**DOI:** 10.1080/23802359.2020.1778567

**Published:** 2020-06-17

**Authors:** Olga V. Bondareva, Ahmad Mahmoudi, Semen Yu. Bodrov, Evgeny A. Genelt-Yanovskiy, Tatyana V. Petrova, Natalia I. Abramson

**Affiliations:** a Department of Molecular Systematics, Zoological Institute RAS, St. Petersburg, Russia; b Department of Biology, University of Urmia, Urmia, Iran

**Keywords:** Mitochondrial genome, NGS, phylogenetic trees, *Ellobius*

## Abstract

The subterranean voles of the genus *Ellobius* are species of subfamily Arvicolinae well adapted to underground life. In this paper, we report the assemblies of complete mitochondrial genomes for three mole voles from genus *Ellobius* – northern mole vole *Ellobius talpinus* (16,376 bp), transcaucasian mole vole *E. lutescens* (16,540 bp), and southern mole vole *E. fuscocapillu*s (16,388 bp). Each of three mitogenomes encode for 12S and 16S rRNAs, 22 tRNAs, 13 protein-coding genes, and D-loop in the characteristic arrangement of subfamily Arvicolinae (Rodentia: Cricetidae). This study verifies the evolutionary status of subgenera *Bramus* and *Ellobius* within the genus *Ellobius* at the molecular level. The mitochondrial genome would be a significant supplement for the *Ellobius* genetic background. The three *Ellobius* species formed a monophyletic group with the high bootstrap value (100%) in all examinations.

Mole voles, of the genus *Ellobius* Fisher, 1814 occupy a wide range of arid and semiarid lands of Eastern Europe, Ural, Middle Asia, West Siberia, Transcaucasia and Southwest Asia (Carleton Michael and Musser 2005; Shenbrot and Krasnov 2005). All species of the genus are highly specialized to underground life style. The genus consists of two subgenera: nominotypical subgenus *Ellobius* Fischer, 1814 with three morphologically cryptic small-sized species: northern mole vole *E. talpinus* Pallas, 1770, eastern mole vole *E. tancrei* Blasius, 1884 and Alay mole vole *E. alaicus* Vorontsov et al., 1969 and subgenus *Bramus* Pomel, 1892 (*Afghanomys* is junior synonym) with a two larger species *E. fuscocapillus* Blyth, 1843 and *E. lutescens* Thomas, 1897. Here, we report the structure of the complete mitochondrial genomes of three species of mole-voles from both subgenera: *E. talpinus*, *E. fuscocapillus*, and *E. lutescens.*

The specimens of *Ellobius talpinus* were trapped in Sarkel, Tsimlyansk Reservoir, Rostovskaya oblast’, Russia (GPS coordinates: 47.681613, 42.144175); *E. lutescens* (13ES-AM) in Daran, Isfahan province, Iran (GPS coordinates: 32.986888, 50.432880). *Ellobius fuscocapillus* (21NK-AM) were collected in Tukur, Shirvan, North Khorasan province, Iran (GPS coordinates: 37.634880, 57.764854).

The tissue and DNA of the specimens were deposited in the Theriology tissue collection of the Zoological Institute RAS, Saint Petersburg, Russia, under the numbers 1924 (*E. talpinus*), 4905 (*E. lutescens*), and 4907 (*E. fuscocapillus*). Total genomic DNA was isolated from the muscle tissue and stored in −20 C. Paired-end sequencing (2 × 75 bp) was performed in an Illumina HiSeq 4000 sequencer in the Genomics Core Facility of Skolkovo Institute of Science and Technology. Sequence reads were submitted to NCBI SRA Database under the numbers SAMN13341616 (*E. talpinus*), SAMN13341617 (*E. lutescens*) and SAMN13341618 (*E. fuscocapillus*).

Raw reads were cleaned by removing reads containing Illumina adapters, overrepresented sequences and low quality reads (<Q20) using Trimmomatic v0.39 (Bolger et al. 2014). Remaining reads were assembled using SPAdes version 3.10.1 (Bankevich et al. 2012). The assembled mitogenome sequences were annotated using the MITOS (Bernt et al. 2013), and annotations were then corrected manually.

The assembled complete mitochondrial genomes are typical for the subfamily Arvicolinae yet demonstrate slight variation in sequence length for mole voles: the mitogenome of *E. talpinus* is 16,376 bp long sequence. *Ellobius lutescens* is 16,540 and *E. fuscocapillus* – 16,388 bp **(**GenBank accession No MT483993, MT483992, and MT483991, respectively). For all three mitogenomes, the complete sequences of 13 protein-coding genes (PCGs), two ribosomal RNA genes (*rrnL* and *rrnS*), 21 transfer RNA genes (*tRNAs*), and a putative control region were obtained. The gene order and organization are consistent with other mitochondrial sequences of arvicolines. Mitochondrial genomes are GC-rich compared with other Arvicolinae mitogenomes: 35.8%, 38.3%, and 36.8% for *E. fuscocapillus*, *E. lutescens* and *E. talpinus*, respectively. The nucleotide composition also varied across species: *E. fuscocapillus* keep A, G, C, and T as 30.9%, 13.0%, 27.4%, and 28.7%, respectively; *E. lutescens*: 28.4% T, 31.4% A, 26.9% C, and 13.5% G; *E. talpinus*: 27.7% T, 30.2% A, 28.7% C, and 13.5% G. The GC-skew of genomes were −0.32, −0.33 and −0.34 for *E. fuscocapillus*, *E. lutescens*, and *E. talpinus*, respectively. Nine genes (*nad6* and eight tRNAs) were oriented in the reverse direction, whereas the others were transcribed in a forward direction.

All three mitogenomes harbor identical 73 bp overlapping sequences in 10 regions. The longest overlap is 43 bp in length and located between *atp8* and *atp6*. All tRNAs have the typical cloverleaf structure, which is similar to those reported in most animal mitogenomes.

The initial codons for 13 PCGs of *E. fuscocapillus*, *E. lutescens*, and *E. talpinus* were the canonical putative start codons ATN (particularly ATG for *cox1, cox2, atp8, atp6, cox3, nad4l, nad4, nad6*, and *cob*); ATT for *nad2* and *nad5* (*E. fuscocapillus*); ATA for *nad3* (*E. fuscocapillus* and *E. talpinus*) and *nad5* (*E. fuscocapillus* and *E. lutescens*). The only exception was the *nad1*, which started with GTG codon for all three species. The typical termination codons (either TAA or TAG) were observed in all PCGs.

Based on the alignment of complete mitochondrial genomes, the Bayesian inference method implemented in MrBayes 3.2.6 (Ronquist et al. 2012) was used to reconstruct the phylogenetic relationships between three *Ellobius* species and other voles, for which complete annotated mitochondrial genome sequences are available at NCBI Nucleotide database. The result of phylogenetic reconstruction is presented in Figure 1. The monophyly of the tribe Ellobiusini is highly supported and the evolutionary status of subgenera *Ellobius* (*E. talpinus*) and *Bramus* (*E. lutescens* and *E. fuscocapillus*) within *Ellobius* is verified at the molecular level. Therefore, mitochondrial genomes would be a significant supplement for the *Ellobius* genetic background. Reported complete mitogenomes of Arvicolinae confirmed monophyly of the subfamily and tribes Clethrionomyini and Dicrostonychini, but available mitogenomes are still insufficient in number for uncovering phylogenetic relationships among the tribes of the subfamily.

**Figure 1.**
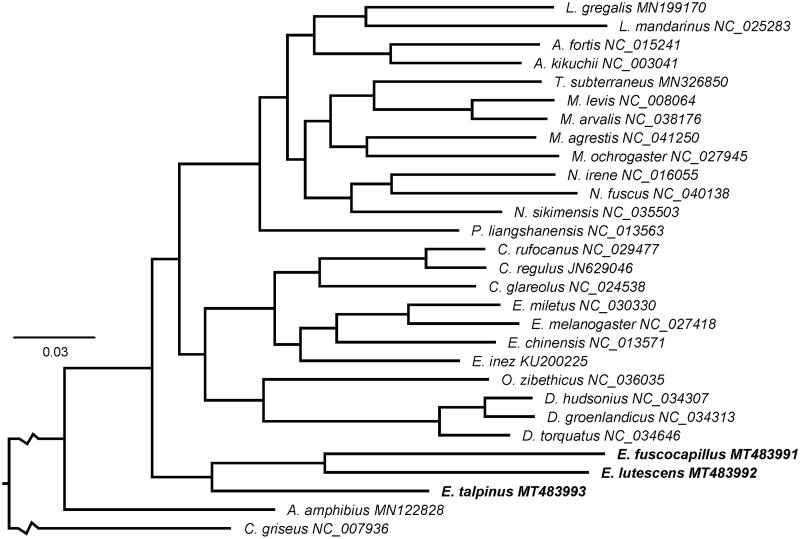
Bayesian tree inferred from the complete mitochondrial alignment. All nodes are supported by a bootstrap greater than or equal to 0.97.
